# Characterization of aerosol particles during a high pollution episode over Mexico City

**DOI:** 10.1038/s41598-021-01873-4

**Published:** 2021-11-18

**Authors:** Giovanni Carabali, José Villanueva-Macias, Luis A. Ladino, Harry Álvarez-Ospina, Graciela B. Raga, Gema Andraca-Ayala, Javier Miranda, Michel Grutter, Ma. Montserrat Silva, David Riveros-Rosas

**Affiliations:** 1 Instituto de Geofísica, Universidad Nacional Autónoma de México (UNAM), Mexico City, Mexico; 2 Facultad de Química, Universidad Nacional Autónoma de México (UNAM), Mexico City, Mexico; 3 Instituto de Ciencias de la Atmósfera y Cambio Climático, Universidad Nacional Autónoma de México (UNAM), Mexico City, Mexico; 4 Facultad de Ciencias, Universidad Nacional Autónoma de México (UNAM), Mexico City, Mexico; 5 Instituto de Física, Universidad Nacional Autónoma de México (UNAM), Mexico City, Mexico

**Keywords:** Environmental sciences, Environmental chemistry, Atmospheric chemistry

## Abstract

More than 7 thousand wildfires were recorded over Mexico in 2019, affecting almost 640 thousand hectares. Most of these fires occurred during the spring season generating dense smoke plumes, impacting urban areas in the central part of the Mexican plateau. From May 10 to 17, 2019, biomass burning (BB) plumes affected Mexico City (MC) and diffused across the basin, producing PM_2.5_ levels ~ 2 times higher than the nation's air quality standards. Average PM_2.5_ concentrations increased sharply from 29.4 ± 7.2 µg m^−3^ to 65.1 ± 13.6 µg m^−3^ when the dense smoke plumes were detected. The higher particle concentration impacted the aerosol optical depth (AOD) as values ~ 3 times greater than the annual mean (0.32 ± 0.12) were measured, which resulted in a 17% loss of global horizontal irradiation (GHI). Under these severe pollution conditions, the visibility (*V*_*a*_) was reduced by ~ 80%. The high incidence of strong absorbent particles, such as soot and tarballs was revealed through electron microscopy and X-ray fluorescence (XRF) analysis. These techniques show chemical similarities between MC aerosols and those from the high-altitude (~ 4010 m. a. g. l.) Altzomoni Atmospheric Observatory, evidencing a strong influence of the BB emissions, suggesting a regional transport of these pollutants.

## Introduction

Atmospheric aerosols can affect the planet's radiation balance by scattering solar radiation, which results in the cooling of the Earth's surface^1,2^. These particles can also indirectly affect climate, based on how they interact with surrounding clouds^3–5^. The direct effects of aerosols on the climate system are much better understood and quantified than the indirect effects. High particulate matter concentrations in the atmosphere, coupled with the dense haze generated by the interaction between intense solar radiation and other atmospheric pollutants, can decrease the global horizontal irradiation (GHI) reaching the surface^6,7^. This reduction in the intensity of solar irradiance occurs mainly due to the influence of carbonaceous aerosols, such as soot that efficiently scatters and absorbs incoming solar radiation, increasing the AOD values and significantly reducing the regional visibility (*V*_*a*_)^6,8^.

The MC metropolitan area (MCMA), formed by adjacent municipalities, and other urban zones from the State of Mexico and Hidalgo, is considered one of the largest emission sources of atmospheric pollutants in the central Mexico plateau^9,10^. Additionally, the MCMA may be affected by the regional transport of BB aerosols mainly in spring (March–May)^11–13^ where dense smoke plumes containing soot have been widely documented^11,12^. Previous studies reported that wildfires in and around the MC basin impact regional air quality^11,13^. High atmospheric pollution (HAP) episodes were very frequent in MC in the 1990s ^15,16^; however, the systematic measures implemented to mitigate urban emissions over the decades have significantly decreased their occurrence.

A quick assessment of the air quality in a given region can be done by measuring *V*_*a*_, which can be calculated from the extinction coefficient (*β*_*ex*_) and estimated from AOD measurements performed by the Aerosol Robotic Network (AERONET)^17^. Likewise, the radiation attenuation due to clouds and atmospheric pollution can be estimated using the European Solar Radiation Atlas (ESRA) model, which calculates solar irradiance under clear sky conditions^18^. Therefore, a correlation between solar radiation measurements (e.g., GHI data) with ESRA calculated values allows estimating solar irradiation losses^18^.

MC was affected by a severe pollution episode in May 2019, detected by the Automatic Atmospheric Monitoring Network (RAMA by its acronym in Spanish) stations. The concentrations of PM_2.5_ presented a rapid and dramatic increase from May 10 to 17. This HAP episode significantly affected the *V*_*a*_ and degraded the air quality in MC, a fact that alarmed MC’s authorities, who decreeing an environmental contingency to mitigate their impacts. Although most of the airborne pollutants are assumed to be generated by forest fires and the ubiquitous anthropogenic activities within the MC basin, the detection of dense smoke plumes at the high-altitude Altzomoni Atmospheric Observatory (AAO) suggests that BB aerosols can be transported on a regional scale.

In this study, aerosol optical properties were measured during the HAP period (May 10 to 17, 2019) to evaluate the effect of high PM_2.5_ concentrations on the GHI and AOD values, as well as in the *V*_*a*_ degradation. Additionally, a morphological and chemical analysis of the aerosol samples collected in MC and the AAO was performed with transmission (TEM) and scanning (SEM) electron microscopes coupled with X-ray analyzers^19–21^. This analysis allows identifying the primary sources of aerosol particles that significantly reduced air quality in MC.

## Methods and instrumentation

A direct comparison of the locally emitted particles vs. the long-range transported aerosol particles into the MCMA was assessed at the high-altitude AAO^22–24^. Given that the AAO is ~ 4010 m a.g.l. (and 1500 m above MC)^22,25^, it allows the collection of aerosol particles within the free troposphere (FT) and the mixed layer (ML)^22,26^. Due to its location and its research facilities, the AAO is part of the Network for the Detection of Atmospheric Composition Change (NDAC) since 2015^22,26,27^. Studies conducted at AAO have focused on volcanic emissions^28–30^, ML pollutants^22,23^, and mixed-phase cloud formation^25^.

### Sampling sites

#### Mexico city

The MCMA consists of 60 agglomerated municipalities^14^. It is considered the largest megalopolis in North America with more than 20 million inhabitants residing in ~ 1500 km^2^. MC located in a subtropical zone is affected by multiple fires during the dry-warm (DW) season, from March to May^11^. The sampling site was placed on the roof of the Institute of Atmospheric Sciences and Climate Change (ICACC) building at the UNAM campus located in southern MC (19°20′N, 99°10′W, 2440 m a.s.l.).

**Figure 1:**
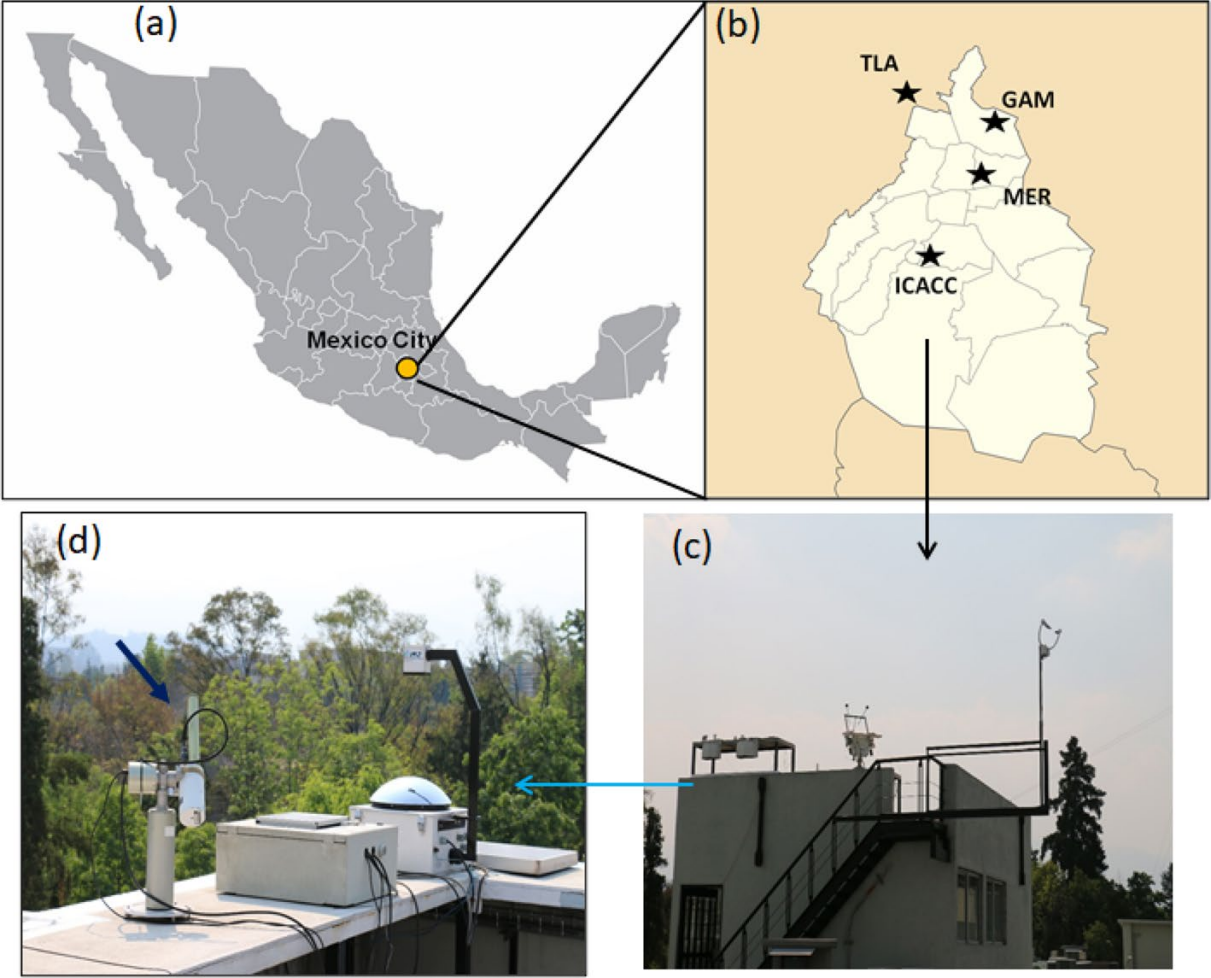
(**a**) Map of Mexico and the geographic location of the MC. (**b**) Map of MC basin and the location of ICACC aerosol observation site. (**c**) AERONET site at the OSR and (**d**) picture of the CIMEL-318 sunphotometer on its measurement platform.

Figure 1a shows the MC location in central Mexico. Although PM_2.5_ concentrations were measured at four RAMA stations (Fig. 1b), the primary sampling site was located at the ICACC station (Fig. 1c), which has a Solar Radiation Observatory (OSR) with a suitable platform for the installation of the CIMEL sun photometer (Fig. 1d).

#### Altzomoni atmospheric observatory

The sampling was carried out at the AAO (19.117°N, 98.654°W), located approximately 60 km southeast of MC, 70 km northeast of Cuernavaca, and 50 km west of Puebla (Fig. S1). Its proximity to the active Popocatepetl volcano (~ 12 km), makes the AAO a strategic site for studying the impact of volcanic activity on the atmosphere. Altzomoni mountain is generally above the ML from late evening until late morning^22–24^. Ceilometer and radiosonde measurements show that the height of the ML increases rapidly between 11:00 and 13:30 local standard time (LST), with an average growth rate of more than 600 m per hour. After 13:30 LST the growth rate abruptly slows down. Garcia-Franco et al. (2018)^23^ and Whiteman et al. (2000)^24^ concluded that the maximum height reached by the ML is > 3 km a.g.l during the dry-warm season (March–May).

Near pristine conditions are observed at the AAO during the rainy season, whereas BB prevails during the dry season^11,12,31,32^. At the altitude of the AAO surrounding areas are dominated by tall grasses. Due to the ML diurnal growth, the AAO site is affected by anthropogenic emissions generated in the MCMA^22,33^. Additionally, it may also be affected by ash and volcanic gases generated by Popocatepetl degassing and explosions^22,28,30,34^. Remote sensing studies of the volcanic plumes have revealed that SiF_4_, SO_2_, HCl, HF, H_2_S, CO, CO_2_, and H_2_O are commonly emitted ^28,30,35^.

### Aerosol sampling

Atmospheric particles analyzed in this study were sampled at the ICACC and the AAO sampling sites. Aerosol particles were collected directly on TEM grids (200-mesh, Gilder Cu-grids from Ted Pella Inc. USA). The TEM grids were placed on hydrophobic glass coverslips (HR3-215; Hampton Research) by fixing them with double-sided adhesive carbon tape. Subsequently, the glass coverslips were placed in stages 5 and 6 (cut sizes of 1.0 and 0.56 µm, respectively) of an 8-stage Micro Orifice Uniform Deposit Impactor (MOUDI, Model 100R, MSP Corp.)^36^. The glass plates were fixed onto the MOUDI stages by substrate holders, as reported by Córdoba et al. (2021)^37^. The MOUDI inlet flow was calibrated before sampling to 30 L/min using a Gilibrator air flow calibrator (Sensidyne, Inc., Clearwater, Florida, USA). A four-hour sampling period was selected to avoid particle agglomeration on TEM grids and to allow better analysis of individual particles. After each sampling, the glass coverslips with the TEM grids were stored in sterilized Petri dishes at 4 °C. Additionally, PM_2.5_ samples were collected onto Teflon filters with a MiniVol sampler (AirMetrics) over 24 h periods at a flow rate of 5 L min^−1^.

### Fine particulate matter (PM_2.5_) measurements

Continuous PM_2.5_ concentrations were obtained from the RAMA (http://www.aire.cdmx.gob.mx) using the beta-attenuation methodology. The RAMA air quality network has 30 stations deployed throughout MC. Additionally, the RAMA has a laboratory for equipment maintenance and calibration. PM_2.5_ concentrations for this study were obtained from the following 4 monitoring stations (Fig. 1b):

- Instituto de Ciencias Atmosféricas y Cambio Climático (ICACC) station, located in the south of MC.
- Gustavo Madero (GAM) station, located in the northern-central part of MC (downtown).
- La Merced (MER) station is located in the central-eastern region of MC.
- Tlalnepantla (TLA) is in the north of MC.

### Aerosol morphology and elemental analysis

#### TEM, SEM, and EDS analysis

The electron microscopy analyses were performed at the Central Microscopy Laboratory of the Institute of Physics of the UNAM (IFUNAM). Electron microscope images for morphological characterization were taken in a high-resolution TEM, JEOL JEM-2010F microscope (FasTEM, JEOL, Tokyo, Japan) operated at 200 kV near the Scherrer focus, with a theoretical point-to-point resolution of 0.20 nm and a spherical aberration of 0.5 mm. To obtain the aerosol images, copper TEM-grids with particle samples were introduced into the microscope. The images were obtained after reaching the optimum vacuum. The images were recorded with a charge-coupled device (CCD) camera and processed with the GATAN digital micrography system (version 3.7.0, Roper Technologies, Inc., Sarasota, FL, USA). To obtain the micrographs of the soot particles the TEM was operated at high magnifications. SEM images were obtained with a field emission ultra-high-resolution SEM JEOL-JSM-7800F equipped with an Oxford Instruments Energy-dispersive X-ray Spectroscopy (EDS, Oxford Instruments, Abingdon, Oxfordshire, UK) detector and the AZtec 2.1 analysis software. The SEM operating voltages depend on the substrate and fluctuated between 1 and 10 kV. Micrographs obtained with SEM and TEM were analyzed using the ImageJ and Scion 4.0 software, which are public domain. EDS spectra were used to analyze the elemental compositions of particles with diameters from nanometers to micrometers. Expanded electron beams covering whole particles (~ 1 µm) were used. In this way, the average compositions of individual particles were determined. Various particles were measured in the TEM employing magnifications from ~ 2000 × to 5000 ×.

#### X-ray fluorescence (XRF) analysis

A custom-built XRF spectrometer for environmental applications equipped with an X-ray tube with Rh anode operated at 50 keV and 500 µA (Oxford Instruments, Mountain View, CA, USA) was used for PM_2.5_ elemental analysis. The detection system consisted of an Amptek X-123SDD spectrometer with a resolution of 120 eV at 5.9 keV. The Teflon filters (Whatman) with PM_2.5_ samples collected with a MiniVol (AirMetrics) from 12 h at a flow rate of 5 L min^−1^ were placed in the analysis chamber at a high vacuum (10^−6^ torr). The XRF spectrum was collected for 900 s and subsequently integrated with the Quantitative X-ray Analysis System (QXAS). The XRF spectrometer calibration procedure was achieved using thin-film standards (MicroMatter Co., Vancouver, Canada), irradiated for 300 s under the same conditions as the sample analysis. Then, accuracy checks were performed using the NIST standard reference material 2783^38,39^.

### Extinction coefficient and AOD measurements

Measurements of the *β*_*ex*_ were obtained from the database of the University Network of Atmospheric Observatories (RUOA, for its acronym in Spanish) https://www.ruoa.unam.mx^26^. The *β*_*ex*_ is obtained from a Photoacoustic Extinctiometer (PAX) (Droplet Measurement Technologies, Boulder, CO), operated at a flow rate of ~ 1 L min^−1^, which is a sensitive and high-resolution instrument that measures the optical properties of aerosol particles^40^. The PAX uses a 532 nm diode laser to simultaneously measure absorption and scattering coefficients.

AOD measurements were performed with a CIMEL sun-photometer (CE-318) associated with AERONET^41^ and located at the UNAM main campus in southern MC. The CE-318 device is a spectral radiometer (340, 380, 440, 500, 675, 870, and 1020 nm) programmed to automatically track the Sun following scheduled procedures to estimate the AOD. All measured optical parameters can be download from the AERONET database (https://aeronet.gsfc.nasa.gov), as well as the fine- (FM) and coarse-modes (CM) contributions to the total AOD, estimated via the Spectral De-convolution Algorithm (SDA)^42,43^. Cloud-screened (Level 1.5) data, following the methodology described by Smirnov et al. (2000)^44^, was used in this study.

### Estimation of the visibility

*V*_*a*_ is defined as the maximum horizontal distance that the human eye can see. In the present study *V*_*a*_ was calculated from *β*_*ex*_ values by using the Koschmieder equation (Eq. 1)^17^:

1 $$V_{a } = 3.912\,\, \beta_{ext}^{ - 1} ,$$

where *β*_*ex*_ is the extinction coefficient measured at 532 nm.

Additionally, *V*_*a*_ was calculated using Eq. (2) from Baumer et al. (2008)^17^, assuming *β*_*ex*_ independent of height (i.e., along the atmospheric vertical column). This estimate also assumes that all aerosol is located within the mixing layer with a height *Z*_*i*_.

2 $$V_{a } = 3.912\,\, (Z_{i} ) \,\,AOD_{500}^{ - 1} ,$$

where the mixing layer height (*Z*_*i*_) was considered constant with values of 1.0, 1.5, and 3 km taken from Ceilometer measurements. AOD_500_ is the AOD at 500 nm measured by the CIMEL sun-photometer.

### Global solar radiation measurements

Hourly Global Horizontal Irradiation (GHI) was calculated with the global solar irradiance measurements (*I*_*gg*_) from the Observatory of Solar Radiation (OSR) on the Institute of Geophysics at the National Autonomous University of Mexico in Mexico City. The CMP22 radiometer (Kipp & Zonen) samples every 4 s and reports the average every minute. Equation (3) shows the calculation of the solar irradiation.

3 $$GHI = \int_{t1}^{t2} {I_{gg} dt}$$

To determine the influence of atmospheric suspended particles on reduced solar irradiance, the GHI maximum values for every day were correlated with the corresponding PM_2.5_ values. The average daily maximum GHI registered for May was calculated and subtracted from the maximum irradiation values of each day. This allows observing the solar radiation reduction during the HAP days.

Additionally, solar irradiances under cloud-free sky conditions for the ICACC station in MC were calculated with the European Solar Radiation Atlas (ESRA), which is used in the Heliosat-2 model^18^. ESRA is an empirical model based on a climatological monthly means of the Linke turbidity (*T*_*L*_) factor. Values of *T*_*L*_ for different regions of the world can be found on the Solar Radiation Data (SODA) website (http://www.soda-pro.com/).

### Ceilometer measurements

Continuous vertical profiles of the backscattered laser signal were measured with a Vaisala CL31 Ceilometer based on the lidar technique, which uses light pulses sent to the atmosphere from a laser source. Subsequently, the Ceilometer measures the elastically scattered waves returning to the surface^23^. The Vaisala CL31 uses an indium-gallium-arsenide pulsed-diode laser emitting 910 nm pulses at a repetition rate of 10 kHz. The device also uses a single lens for transmitting and receiving light. The maximum detectable cloud-base height of this instrument is 7500 m a.g.l. Backscattered profiles were recorded every 2 to 120 s at a maximum vertical resolution of 10 m^23^.

### HYSPLIT model

The 24-h air-mass back-trajectories arriving at the AAO at the 500, 1000, and 1500 m a.g.l. were calculated using the Hybrid Single-Particle Lagrangian Integrated Trajectory Model (HYSPLIT) from the National Oceanic and Atmospheric Administration (NOAA). The HYSPLIT model allows computing simple air parcel trajectories of air pollutants^45^. The back trajectories for the 4 days of the HAP episode (i.e., May 14–17) were calculated using the Real-time Environmental Application and Display System (READY).

## Results and discussions

### Heavy aerosol pollution episode in Mexico City

**Figure 2:**
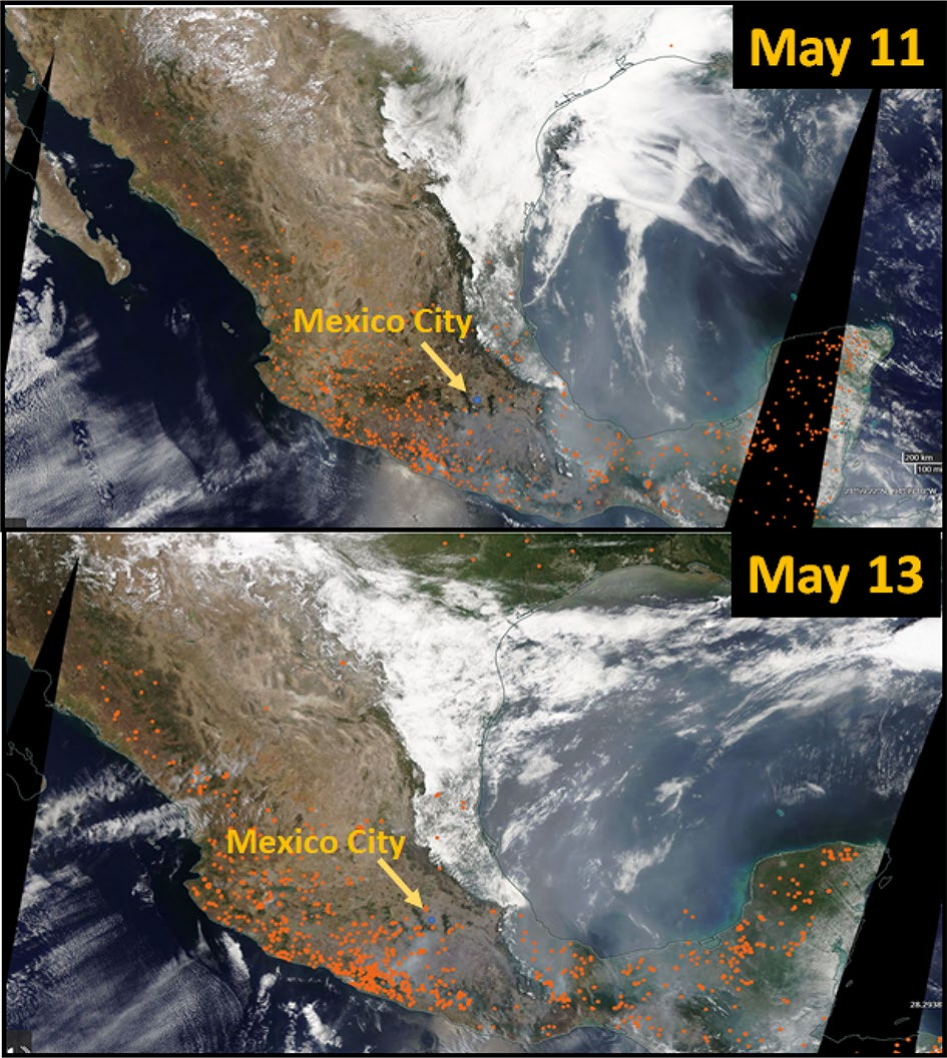
Spatial distribution of active fires (red dots) in Mexico detected by the Visible Infrared Imaging Radiometer Suite (VIIRS) on May 11 and 13, 2019 (Courtesy NASA, https://worldview.earthdata.nasa.gov).

The central part of Mexico during the dry-warm season is dominated by an anticyclonic system, which leads to sunny, warm weather and low precipitation in the MCMA^46,47^. These hot and dry conditions favor the presence of dust and BB emissions from wildfires^11^. Nearly 7410 fires and almost 589,371 hectares burned were reported in Mexico during 2019 (CONAFOR, 2020)^48^. Figure 2 shows the location of active fires in Mexico detected by the Visible Infrared Imaging Radiometer Suite (VIIRS) on May 11, and 13. Fires occur throughout Mexico but are concentrated in the western, central, and southwestern regions. Satellite imagery shows the dense smoke plumes emitted by the wildfires, which reached the central Mexico plateau. There were also a significant number of fires that occurred inside and around the Mexico City basin.

**Figure 3:**
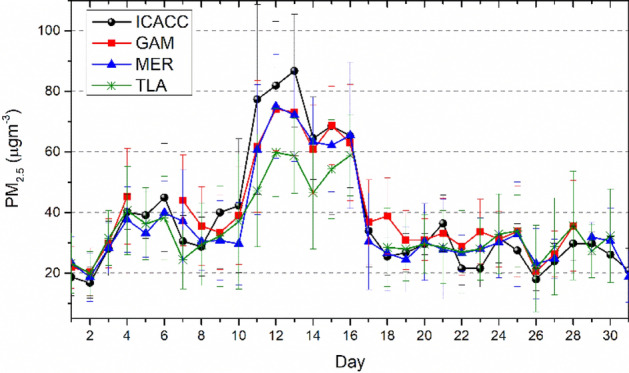
Time series of the 24-hourly means of the PM_2.5_ concentration during May 2019. Data were plotted for the four RAMA stations: ICACC (black), GAM (red), MER (blue), and TLA (green).

Figure 3 shows the 24-h average PM_2.5_ concentration measured during May 2019 in four RAMA stations: ICACC, GAM, MER, and TLA in MC. All stations located in different parts of the MC registered a drastic increase in 24-h average PM_2.5_ from May 10 to 17. The 24-h PM_2.5_ mean concentration at the ICACC station during HAP days was 65.1 µg m^−3^, which significantly exceeded the Mexican national air quality standards (MNAQS) of 45.0 µg m^−3^. That PM_2.5_ increment during the HAP episode began on May 10 reaching the maximum concentrations on May 12 and 13 with peak PM_2.5_ values of 81.9 and 86.8 µg m^−3^, respectively. Based on the PM_2.5_ concentrations shown in Fig. 3 and taking into account the metropolitan air quality index (IMECA) as a reference, we will classify air pollution into two groups: Poor Air Quality (PAQ) days (May 01–09 and 18–31), when PM_2.5_ concentrations are greater than or equal to the MNAQS (i.e., PM_2.5_ ≤ 45.0 µg m^−3^) and Very Poor Air Quality (VPAQ) days (May 10–17) when concentrations are greater than the MNAQS (i.e., PM_2.5_ > 45.0 µg m^−3^). The average values of PM_2.5_ concentration measured at the ICACC station are shown in Table 1.

**Table 1:** Average values of PM_2.5_ concentration (ICACC measurements), AOD, and *V*_*a*_ during VPAQ days (May 10–17), PAQ (May 01–09 and 18–31), and annual means.

	PM_2.5_ (µg m^-3^)	AOD	V_A_ from β_ext_ (km)	V_A_ from AOD (km) Z_i_ = 1.0, 1.5, and 3.0
VPAQ	65.1 ± 13.6	0.80 ± 0.13	10.3 ± 3.8	4.9 ± 0.1, 7.3 ± 1.2, and 14.7 ± 2.4
PAQ	29.4 ± 7.2	0.43 ± 0.16	60.5 ± 15.1	9.1 ± 3.4, 13.4 ± 5.1, and 27.3 ± 10.1
Annual mean	20.9 ± 13.6	0.30 ± 0.12	–	–

### Aerosol optical depth and visibility

**Figure 4:**
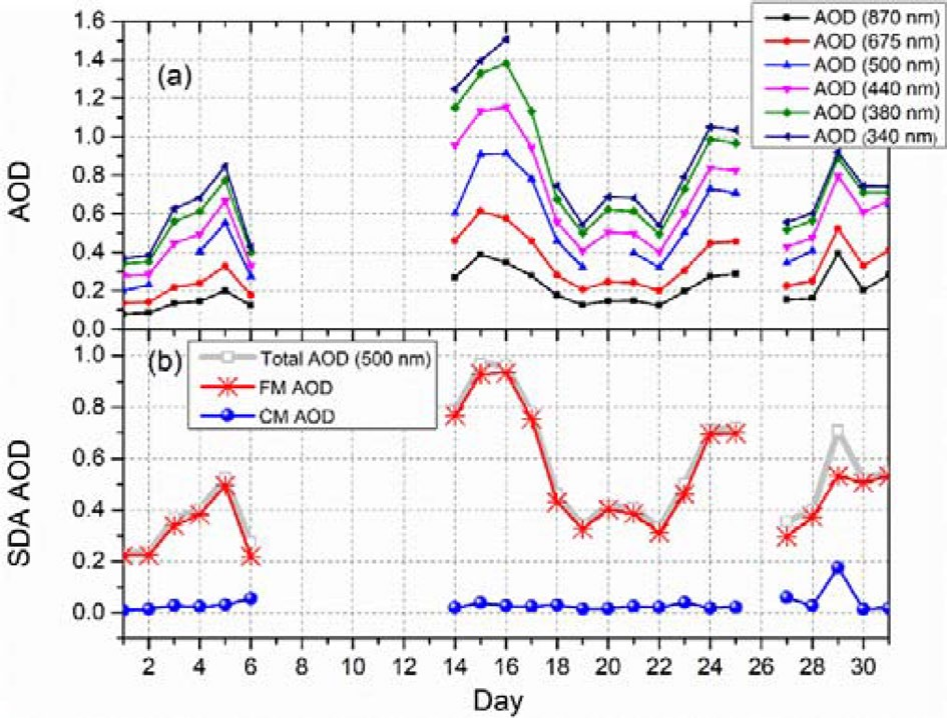
(**a**) Time series of AOD at 6 wavelengths (340, 380, 440, 500, 675, and 870 nm) and (**b**) SDA retrievals of the FM and CM contributions to the total AOD at 500 nm.

Figure 4a shows the average AOD over daytime measurements at 6 different wavelengths (340, 380, 440, 500, 675, and 870 nm) during May 2019, with the highest AOD values observed from May 14 to 18. Due to technical failures, the sensor did not report data the second week of May, including the beginning of the HAP episode. Nevertheless, for the remaining days of the HAP episode (from 14 to 18) high AOD values were observed, with maxima of 0.90, 0.91, and 0.78 measured on May 15, 16, and 17, respectively. These maximum values are ~ 3 times higher than the annual mean AOD (0.32) registered during May 2019. These AOD peaks are caused by the high aerosol loading, evidenced by the PM_2.5_ measurements. Although all aerosols contribute to the AOD increase, the SDA, calculations suggest that the AOD variability in MC is dominated by FM particles. Figure 4b shows the trend of the FM and CM contribution to the AOD (500 nm) measured during May 2019. The FM AOD during the entire month ranges between ~ 0.20 and ~ 0.95, while CM AOD varies between 0.01 and 0.03. Carabali et al. (2017)^10^ reported similar SDA results in MC, demonstrating that fine particles highly contribute to AOD. Similarly, another study of aerosols in the MC during spring 2019 found that fine particles originate mainly from BB and local traffic emissions, while the primary source of coarse particles is dust from re-suspended soil dust^38^.

**Figure 5:**
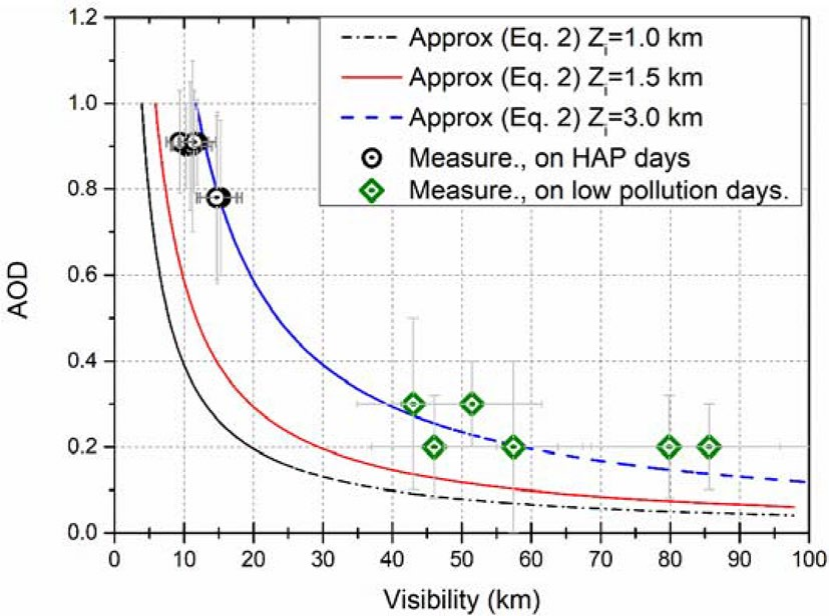
Visibility as a function of AOD values (at 500 nm) from AERONET. Visibility is estimated from *β*_*ext*_ (532 nm) and calculated using Eq. (2).

A remarkable reduction in *V*_*a*_ was also noted in MC during the HAP event. Figure 5 shows the scatter plot of AOD at 500 nm and both *V*_*a*_ calculations (i.e., *β*_*ext*_ and AOD_500_), where a good correlation for Z_i_ = 3.0 km was obtained. The impact of the atmospheric pollution on *V*_*a*_ can be estimated for the 7-days of the HAP period, as seen in Table 1 for 24-h averages, *V*_*a*_ suffered degradation of ~ 80% during the HAP days.

**Figure 6:**
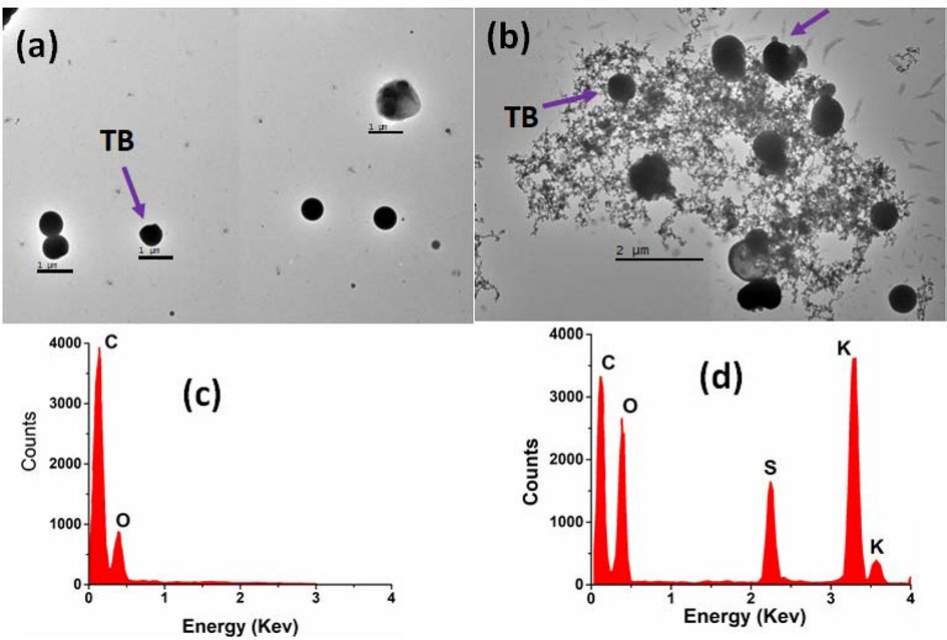
TEM images of the (**a**) TB and (**b**) Soot particles, and EDS spectra of: (**c**) TB and (**d**) soot particles sampled in MC.

TEM analysis of aerosol particles sampled at MC during the HAP episode evidences the presence of soot particles and tarballs (TB) (Fig. 6). TEM micrographs show particles of different sizes and morphologies. For example, Fig. 6a shows spherical particles with diameters (d_p_) < 1 µm. These particles have an elemental composition dominated by a strong C and minor O signal (Fig. 6c), a characteristic composition of TB, a type of particles originating from incomplete combustion of fossil fuels and biomass^49,50^. Although the samples collected showed a high amount of TB particles, it is difficult to know the exact origin of these particles, due to the various sources within the MC. Figure 6b shows the TEM image of TB particles attached to soot agglomerates, whose EDS spectra consist mainly of C, O, and high-intensity K and S signals (Fig. 6d).

### Aerosol effect on global solar irradiance

**Figure 7:**
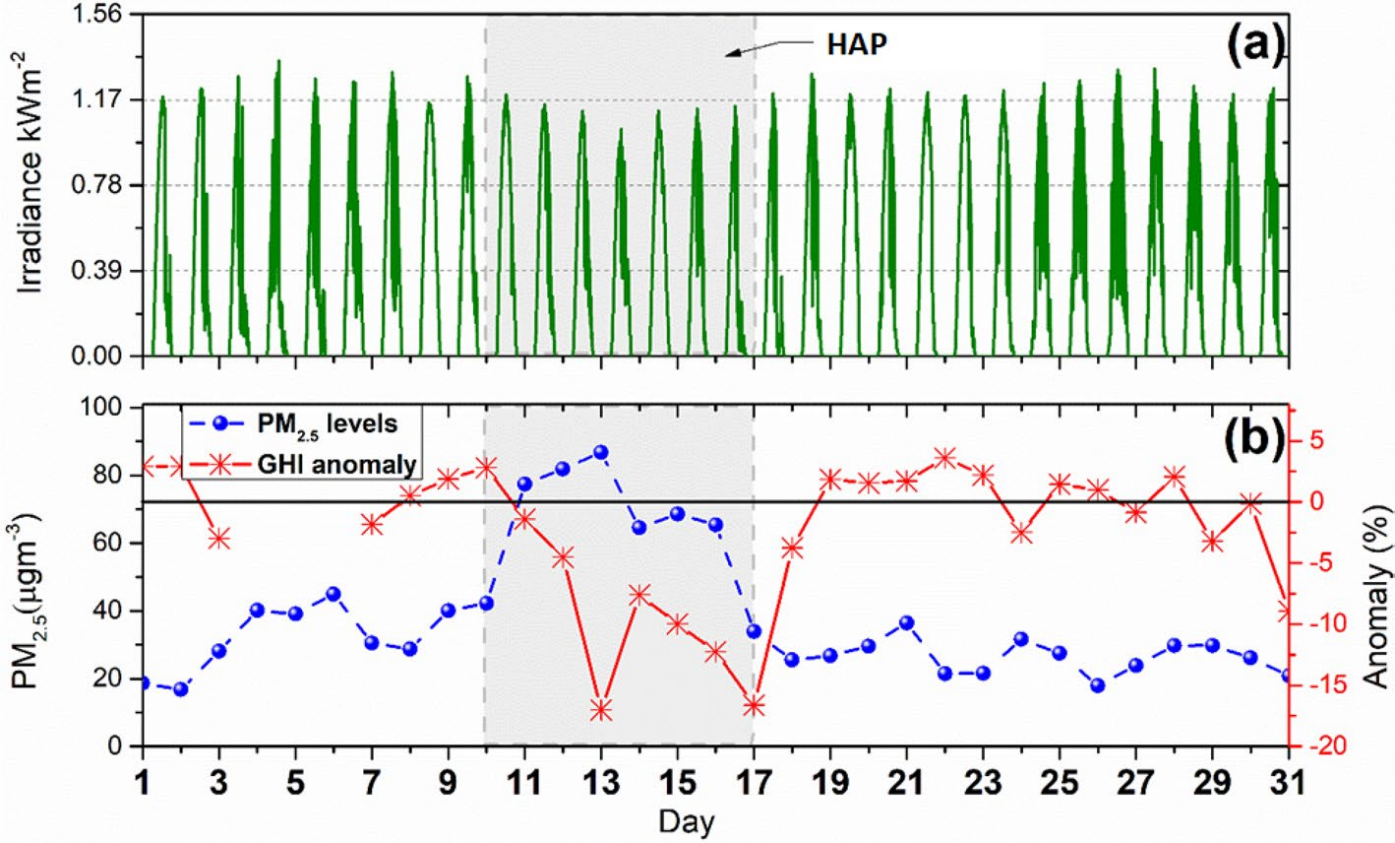
(**a**) Daily GHI measurements and (**b**) hourly GHI percent anomaly (solid red line) compared with the daily mean PM_2.5_ concentration (blue dashed line) measured at the ICACC stations in MC during May 2019.

The influence of aerosols on daily solar irradiation is analyzed in Fig. 7. The observed increase in PM_2.5_ concentrations from 10 to 17 May had an obvious effect on GHI (Fig. 7a, shaded period). The data indicate that GHI gradually decreases as PM_2.5_ levels increased, mainly due to the scattering and absorption of sunlight^51^. The significant reduction in irradiance occurs mainly between 11:00 h and 18:00 h LST (Fig. S2a), when maximum irradiance values are detected, coincides with the highest presence of smoke. To quantitatively estimate the PM_2.5_ impact on the solar irradiance, the maximum daily GHI measurements were subtracted from the monthly mean GHI_m_ value. This difference (∆GHI = GHI − GHI_m_) taken as an anomaly in percent (or departures from the mean monthly value) has a direct impact on visibility. Figure 7b shows the ∆GHI and the PM_2.5_ trends, where it can be observed that the maximum GHI has a significant reduction due to the increase of the PM_2.5_ levels. This high load of aerosol particles during the HAP days resulted in a significant loss of 17% in the GHI. The monthly mean value of the GHI measured experimentally (1130 ± 66 W h/m^2^) fits well with the value calculated theoretically with the ESRA clear sky model^18^ (i.e., 1108 W h/m^2^) that represents the typical GHI value for this month in MC, according to the Linke turbidity value (equal to 4) reported for SODA web services. Figure S2b shows the correlation between these quantities, with a correlation coefficient of 0.54. The negative tilt matches solar irradiation reduction, corresponding with the PM_2.5_ values increasing. The average values of the GHI and its anomaly during the VPAQ and PAQ days of May 2019 are shown in Table S1.

### Aerosol characterization at the AAO

#### Vertical transport of pollutants within the mixing layer

Ceilometer measurements were used to estimate the height of the ML, required to infer the vertical distribution of aerosols. The derivation of the ML height during spring is reliable due to suitable meteorological conditions (low humidity, clear skies, and the absence of precipitation) and high aerosol loading in the atmosphere. Figure S3 shows the resulting profile of the ML height estimates for the MC on May 14, 2019. The sharp decrease in aerosol backscattering between the ML and FT (contrasting colors in Fig. S3a) indicates the boundary between the ML and the FT. The ML time series starts in the early morning hours, with an average height of 900 m a.g.l, until midday when it increases rapidly, reaching heights > 3500 m a.g.l. due to turbulence and dry convective processes. The ML collapses after sunset, as seen in the height decrease around 18:00 LST. ML expansion was also evident with the increase in particle concentration at the AAO. Figure S3b shows the total particle concentration (sizes > 30 nm) measured with a condensation particle counter (CPC) at the AAO. A rapid increase in particle concentration was observed at 11:00 LST, and subsequently, the maximum value was reached at 11:30 LST. Similar results were published by Baumgardner et al. (2009)^22^, who found that particulate matter concentration and other pollutants reach their maximum concentration at mid-afternoon.

#### Aerosol particle types at AAO

Analysis was carried out on 120 particles and based on the morphology and elemental composition as the main criteria, allowing the particle classification into five groups (Table 2): soot, organic, mineral dust, S-rich, and complex secondary particles. Statistical analysis of the spectra showed that 90% of the particles contain C and O, 50% of the particles contain Si, and 30% present S. Fe and Al were also detected although in a low number of particles with weak signals. Copper was not considered for this classification because this element is present in the TEM grids. In this work, the morphology and the elemental analyses were the main factors used to identify soot (chain aggregates) and TB (spherical particles) present in almost all the TEM-grids analyzed.

**Table 2:** Aerosol groups observed at the AAO.

Particle group	Particle type	Elemental composition	Particle morphology
Soot	Soot or black carbon (BC)	Strong C signal in EDS spectra Minor O and S	Particle aggregate, chains formed by nanometric carbon spherules
Mineral dust	Mineral	EDS spectra are dominated by the Si signal. Particles containing Al and Fe. Minor signals of K, S, C, and O are observed	Compact and irregular particles
S-rich	S-rich: mineral, with Ca, (C, O, S)-rich particles and Si	Can appear forming CaSO4 particles, S-rich water droplets, and with Si (volcanic origin)	Some have crystalline morphology, other have very irregular shapes. These aerosols are sensitive to a strong electron beam
Organic	TB	Mostly tarball particles. Intense C signal followed by a low signal of O	Spherical particles
Secondary		In this group, all particles present an S signal. They contained C, O, S, and minor K	Irregular shape particles, susceptible to beam damage. Some Ca-S particles mix with mineral, and some mix with S-rich and K-rich particles

#### Soot

**Figure 8:**
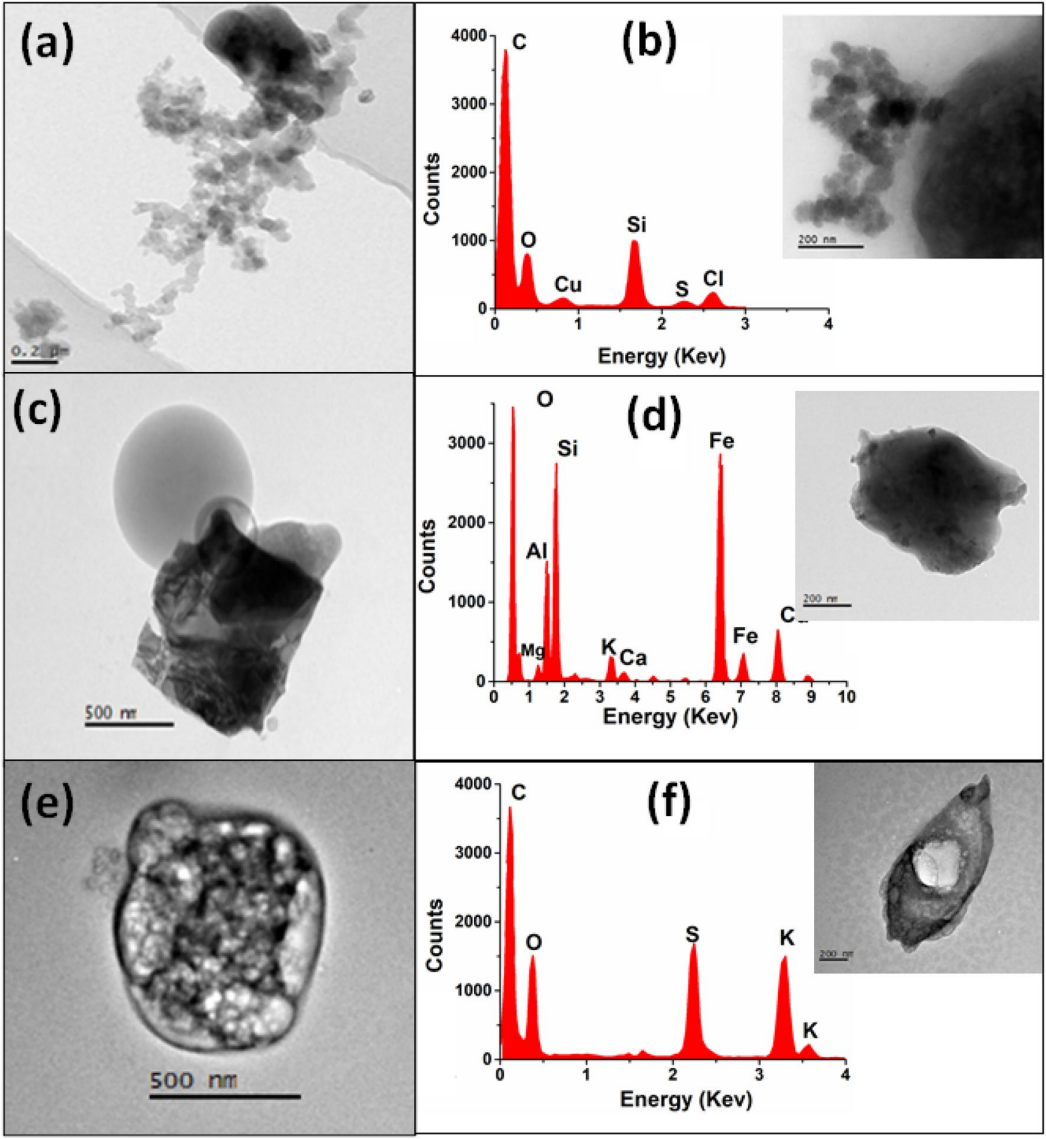
High-magnification TEM micrographs and EDS spectra for (**a**) and (**b**) soot aggregates; (**c**) and (**d**) mineral dust; (**e**) and (**f**) S-rich particles.

Figure 8 shows TEM images of individual soot particles sampled at the AAO site within the FT (i.e., 12:00–05:00 h) during the HAP days. All images show chain-like agglomerated structures of nano-sized primary spherical particles with d_p_ < 60 nm, a typical structure present in soot originated in combustion processes^52^. Those soot particles sampled at the AAO could probably have been produced by BB events or were transported by the ML convective process. The soot particle in Fig. 8a has a d_p_ of ~ 1.7 µm, while the particle in Fig. 8b with a d_p_ ~ 0.7 µm is attached to the bigger one and more compact aerosol. EDS spectra in Fig. 8b consist mainly of three peaks; the most intense is the amorphous carbon signal (~ 0.28 keV), Si, and oxygen (~ 0.53 keV). However, other peaks observed in the EDS (i.e., S and Cl) show the mixed state of the soot. Figure S4 shows an SEM image and the EDS elemental composition mapping of soot particles sampled at the AAO within the ML. The EDS map shows the presence of Si, Al, K, Ca, and Fe, homogeneously distributed throughout the particle which indicates that this particle is an aged aerosol. The high percentage of Si, Al, and O suggests the presence of material with a geological origin that resulted from the resuspension of soil dust.

#### Mineral dust particles

Mineral dust aerosol at the AAO comes mainly from the resuspension of soils and probably from rocks eroded by the wind. These particles with d_p_ < 600 nm (Fig. 8 c and d) show compact shapes and are mixed with other inorganic materials. Figure 8d shows the elemental composition of a mineral particle where a high Si signal is observed, which dominates the composition of mineral particles in this region. Additionally, the mineral particles were found to be mixed with small amounts of aluminosilicates, iron-rich dust, K, and minor Ca. These elements could show the presence of feldspars whose main source could be the erosion of the rocks or could be the result of volcanic emissions^53^. The volcanic ash emitted by the Popocatepetl is one of the main components of the soils that surround the AAO. Although during the sampling days there was no direct influence of the volcanic plumes, the soils surrounding the AAO are covered with material emitted previously. The elemental EDS map in Fig. S5 for a dust particle shows a homogeneous distribution of Si, Al, Mg, and Fe which demonstrates the geological origin of that particle. The presence of C and K with a uniform distribution also indicates that this particle is covered with organic material possibly originated from BB.

#### S-rich particles

Sulfur is one of the most frequently observed elements in the EDS spectra of particles measured at the AAO. The S present in the particles mainly comes from three sources: gas emissions from the Popocatepetl volcano, anthropogenic emissions from MC, and BB emissions. Volcanic emissions are the most important due to the proximity of the AAO to the volcano, which is known as one of the largest sulfur dioxide (SO_2_) sources in the world^28,29^. Volcanic plumes consist of gases and sub-millimeter particles^28,29^. Figure 8e shows a TEM image of an individual S-rich aerosol, and Fig. 8f presents the EDS of this type of particle, both sampled at the AAO during HAP days. S-rich particles present differents morphologies with d_p_ < 800 nm. Some particles presented crystalline structures^54^, but most of them suffered decomposition or evaporation as the microscope beam hit the surface. These particles were beam-sensitive and undergo some changes in their shape during analysis.

#### Tarballs

**Figure 9:**
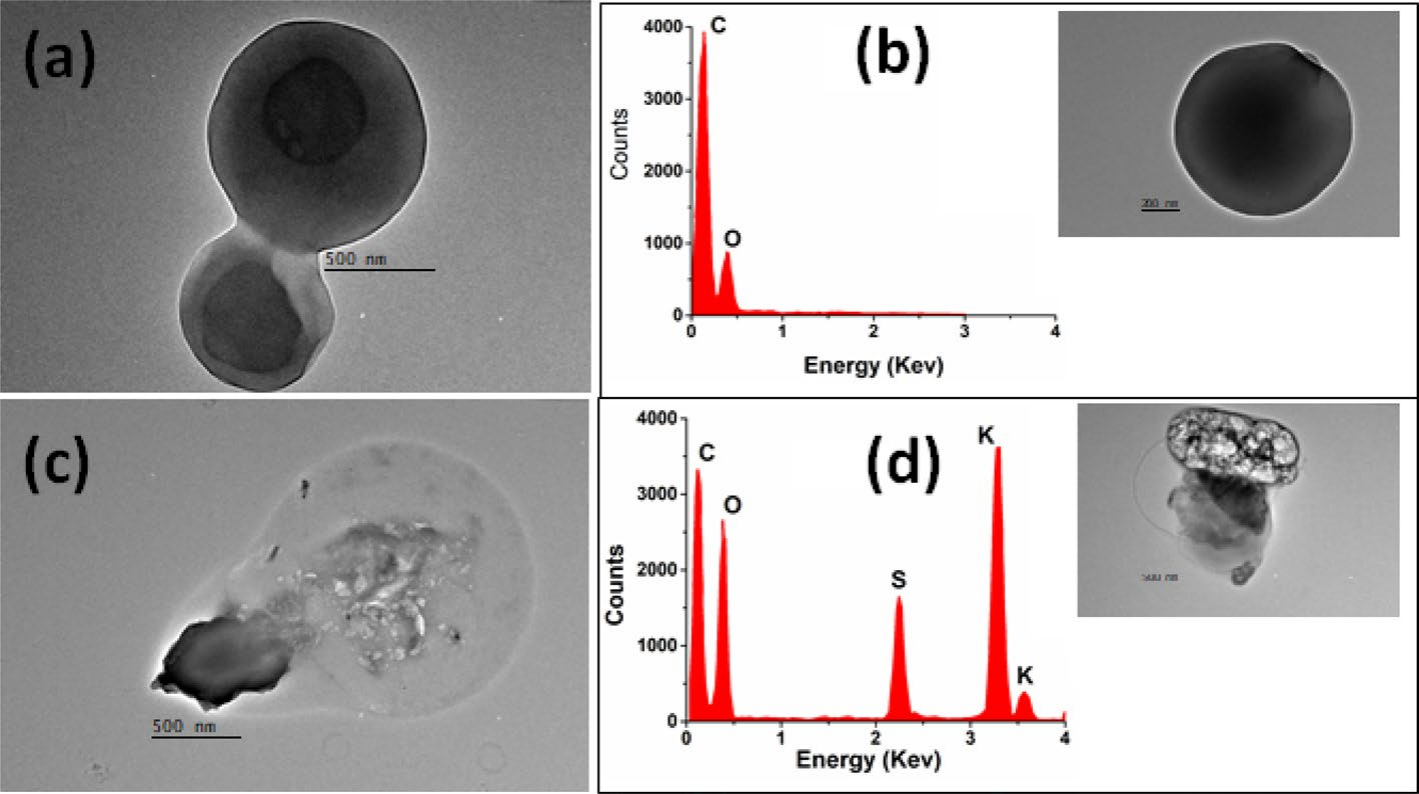
High-magnification TEM micrographs and EDS spectra for: (**a**) and (**b**) Tarballs; (**c**) and (**d**) Secondary aerosols particles.

Figure 9a shows a TEM image of the spherical organic particles with d_p_ < 600 nm, which was common in all aerosol samples collected at the AAO site. Those TB are particles with a special morphology (near-spherical) and composition (carbonaceous material) are quite abundant in biomass smoke plumes^50,55,56^. These particles in the Altzomoni mountain could have two origins: BB and anthropogenic urban emissions. Intense C peak in the EDS spectra confirmed the occurrence of TB at the AAO during the HAP episode (Fig. 9b).

#### Complex secondary particles

A significant number of secondary aerosol particles were also measured at the AAO. These particles are characterized by having complex elemental compositions and very irregular shapes (with d_p_ between 0.5 and 1.6 µm). Figure 9c shows the TEM micrograph of a secondary particle whose morphology was affected by the incident beam of the microscope, and Fig. 9d presents a typical EDS spectrum of this type of particle where S and K signals predominate. The main sources of these particles can be anthropogenic emissions in urban areas and BB emissions. It was observed that most of these types of particles were affected by the strong electron beam of the TEM, evidencing the evaporation of some volatile compounds, which induced changes in their shape.

Most of the particles analyzed in this study were internally mixed, which evidences the presence of aged particles. However, it was also observed aerosol assemblies externally mixed, during the FT hours. Figure S6 shows an example of an externally mixed particle with a soot aggregate surrounded by other particles with different compositions (mainly S). The inset in Fig. S6 is a high magnification image of an internally mixed soot coated with S and K.

#### Elemental composition

**Figure 10:**
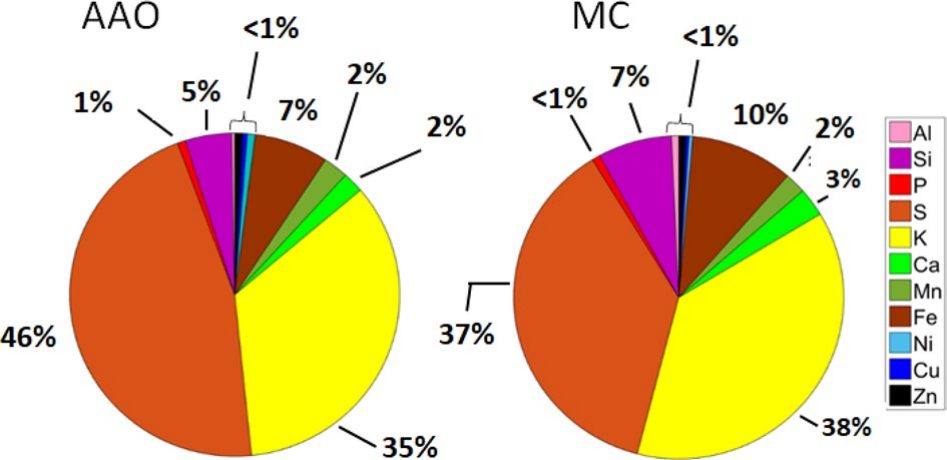
Pie charts of the elemental composition (determined by XRF) of atmospheric particles sampled in two different sites (**a**) AAO and (**b**) MC during the HAP event.

A comparison of the elemental composition of PM_2.5_ particles sampled at the AAO and MC is shown as pie charts in Fig. 10. The main elements detected with the XRF analysis were Al, Si, P, S, K, Ca, Mn, Fe, Ni, Cu, and Zn. Figure 10a shows the elemental analysis of the particles sampled at the AAO, where the composition is dominated by S and K with 46% and 35%, respectively. The abundance of these elements suggested an important contribution of the emissions from wildfires, mainly the K, which is a tracer of BB^57,58^. The existence of dust-like aerosol could be evidence by the presence of Si and Fe with significant contributions of 5% and 10%, respectively, in addition to minor contributions of Al, Mn, and Ca. Although the AAO is located in a remote rural area, small amounts (< 1%) of Ni, Cu, and Zn were measured. These elements could be related to anthropogenic sources close to the sampling site (e.g., chimney of the TV-broadcast antenna facility). Figure 10b shows the elemental composition of particles sampled in MC, which presents values very similar to those measured at the AAO, i.e., with S and K as the elements contributing more to elemental composition with percentages of 37% and 38%, respectively. Although S can be the product of BB, it can also come from other anthropogenic sources (i.e., motor vehicles), being the main element responsible for the production of secondary aerosol particles^58,59^. Mineral-dust aerosol is generally a significant component during the dry-warm period^33,60^. The presence of Fe and Si with percentages of 10% and 7%, respectively, is the primary evidence of mineral dust particles. That mineral fraction in MC is partially a result of the MC semi-arid areas (e.g., former Texcoco and Chalco lakes), arid hills, and unpaved roads within the MC basin^38,61^. The significant Ca contribution could be due to fly ash emissions from two sources: construction activities and soil-dust resuspension^38^. Furthermore, the trace elements with percentages less than 1% (Ni, Cu, and Zn), have been associated with the emissions generated by high traffic in the urban area. For example, most of the Zn detected could originate from the wear of vehicle tires, while Ni and is a typical element in motor fuel additives^38^. The elemental compositions of the particles at both sites (AAO and MC) do not show significant differences. This similarity in the percentage composition could result from the influence of aerosols emitted in the MC, which are transported to the AAO by advective processes in the ML^22^ and orographic forcing. Note that the sampled PM_2.5_ for this analysis was collected every 24 h, making it impossible to separate the FT and ML periods. Excluding the organic materials (not measured in this study), the elemental composition of the aerosol at both sites was dominated by S and K, contributing > 75%, which evidence the strong influence of BB emissions. The present results are consistent with those reported by Decarlo et al. (2008)^10^, who observed elevated sulfate at the higher altitudes above MC. Similar studies of aerosol chemical composition conducted at other high-altitude sites in different parts of the world (in the absence of BB emissions) reveal that aerosols in the FT contain a high fraction of sulfates^10,62,63^.

#### Air masses back-trajectories

BB smoke is one of the main atmospheric components affecting air quality and climate in Mexico due to massive plumes that can travel thousands of kilometers downwind^11,12^. Tracking of these plumes is only possible through satellite measurements, e,g, by the VIIRS radiometer^64^. The HYSPLIT model was used to identify the origin of the air masses reaching the sampling sites. Figure S7a–d show the HYSPLIT back-trajectories computed at different heights (100, 500, and 1000 m a.g.l.) during the HAP episode on May 13, 14, 16, and 17, respectively. The HYSPLIT simulations show that during the HAP episode the air masses mainly originate in western and southwestern Mexico where most of the active fires are concentrated (see fire distribution in Fig. 2) to reach the AAO. However, these air masses also cross over Morelos State, where the cities of Cuautla and Cuernavaca are located, possibly indicating not only volcanic but also urban emissions in the air mass reaching the AAO. Only on May 17, there was a possible transport from north of the AAO, carrying particles emitted in MC.

## Conclusions and discussions

This study successfully combined remote sensing techniques, in-situ measurements, and laboratory analysis to characterize the atmospheric aerosol in MC and the AAO during the HAP period in May 2019. The high levels of PM_2.5_ measured by the RAMA stations throughout the MCMA, evidenced the large amount of aerosol particles. Along May 2019 there was poor air quality, which got significantly reduced with the spread of the wildfires emissions. AERONET results suggest that the extinction of solar radiation in MC was mainly dominated by fine-mode particles. The GHI measured during the HAP was 17% lower than those calculated with the ESRA clear-sky model. It is possible that the high particle loads and dense smoke plumes contributed to the reduction in GHI, which affected both the horizontal and the vertical visibility during the VPAQ days. Although mineral dust particles and other sulfur-rich particles were observed in the collected aerosol samples, the predominant species were soot and TB. The latter two particles were observed simultaneously in the MC and the AAO. Likewise, although the XRF elemental composition of the particles shows similarities in both sampling sites, the higher percentage of S at the AAO particles should be noted. We believe that the higher percentage of S at the AAO suggests a contribution from the regional transport of particles in the FT.

## Supplementary Information

Supplementary Information.
