# Supplementary material for: Characterization of aerosol particles during a high pollution episode over Mexico City
Source: Sci Rep. 2021 Nov 18;11:22533. doi: 10.1038/s41598-021-01873-4 (PMC8602652; doi:10.1038/s41598-021-01873-4)
Supplement: Supplementary file 1 — Supplementary Information. [file 41598_2021_1873_MOESM1_ESM.docx]

**Characterization of aerosol particles during a high pollution episode over Mexico City**

Giovanni Carabali ^1*^, José Villanueva-Macias ^1,2^, Luis A. Ladino ^3^, Harry Álvarez-Ospina ^4^, Graciela B. Raga ^3^, Gema Andraca-Ayala ^3^, Javier Miranda ^5^, Michel Grutter ^3^, Ma. Montserrat Silva^3^, and David Riveros-Rosas ^1^.

^1^  Instituto de Geofísica, Universidad Nacional Autónoma de México (UNAM)

^2^ Facultad de Química, Universidad Nacional Autónoma de México (UNAM)

^3^ Instituto de Ciencias de la Atmósfera y Cambio Climático, Universidad Nacional Autónoma de México (UNAM)

^4^ Facultad de Ciencias, Universidad Nacional Autónoma de México (UNAM)

^5^ Instituto de Física, Universidad Nacional Autónoma de México (UNAM)

* Corresponding author: [carabali@igeofisica.unam.mx](mailto:carabali@igeofisica.unam.mx)

**Supplementary figures**

**
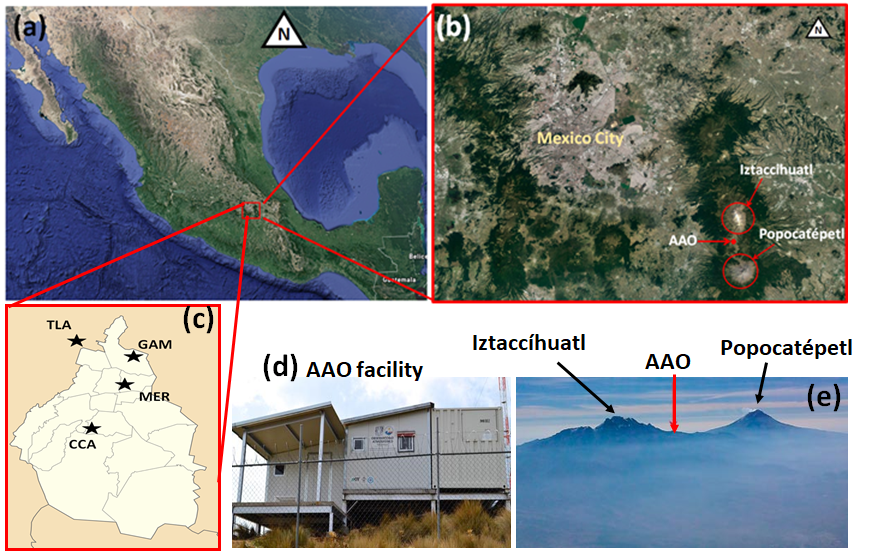
**

**Fig. S1**. The geographical area under study. (a) Map of Mexico with an inset indicating the sampling site, (b) Satellite image showing the location of MC, the AAO, and the nearby volcanoes, (c) Location of the four RAMA stations in MC, (d) The AAO facility, and (e) A panoramic view of the Popocatepetl and the Iztaccihualt volcanoes.


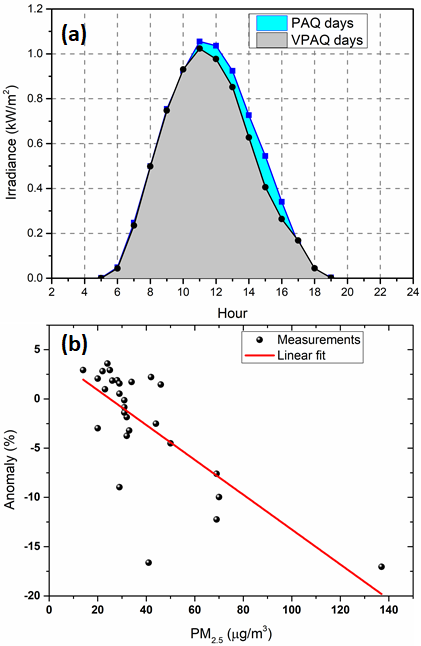


**Fig. S2**. (a) Measurements of daily GHI during the VPAQ and PAQ days. (b) Correlation between the GHI anomaly and PM_2.5_ concentrations.

**
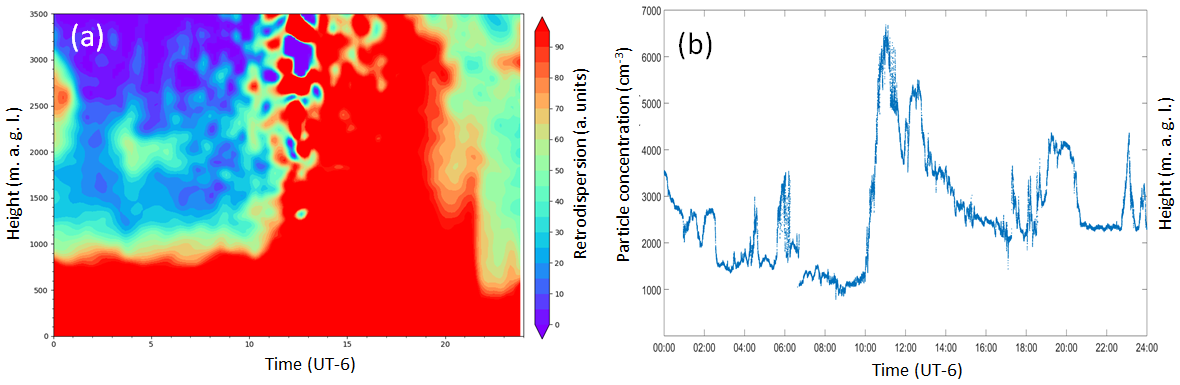
**

**Fig. S3**. (a) Backscatter profile from Ceilometer data measured in MC on May 14, 2019, and (b) particle number concentrations at AAO during the HAP episode.


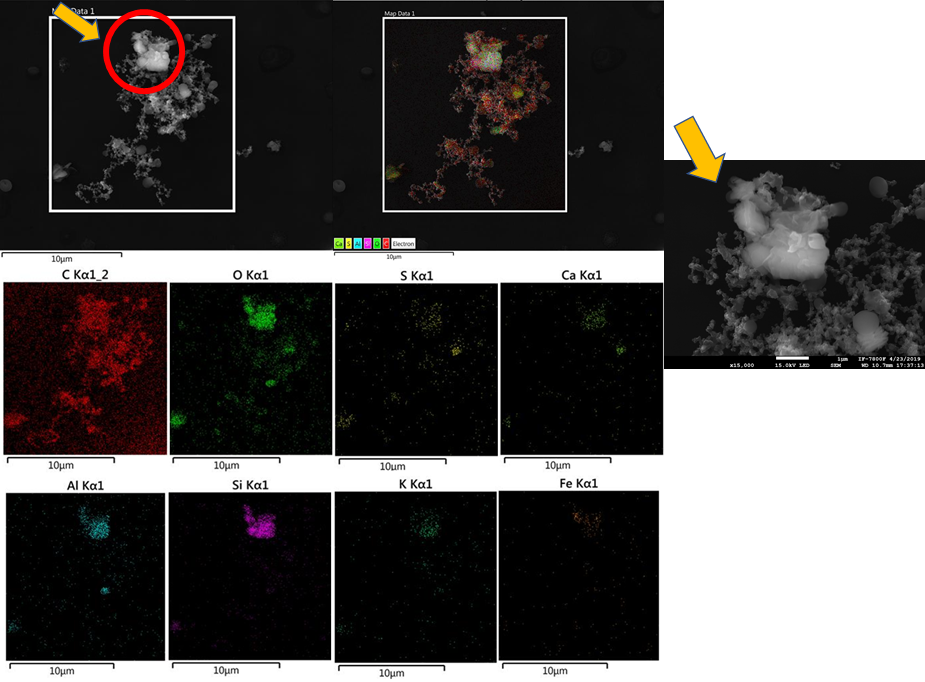


**Fig. S4**. EDS elemental mapping of soot agglomerate sampled at the AAO during the HAP episode.


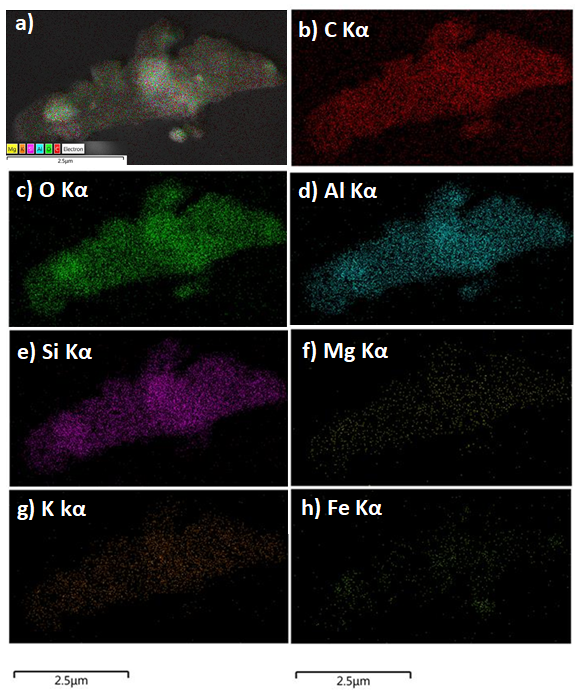


**Fig. S5.** EDS elemental mapping of individual mineral dust sampled at the AAO during the HAP episode


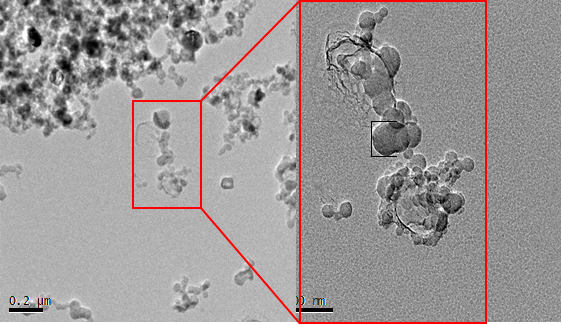


**Fig. S6.** Example of an externally mixed particle with a soot aggregate surrounded by other particles with different compositions (mainly sulfur).


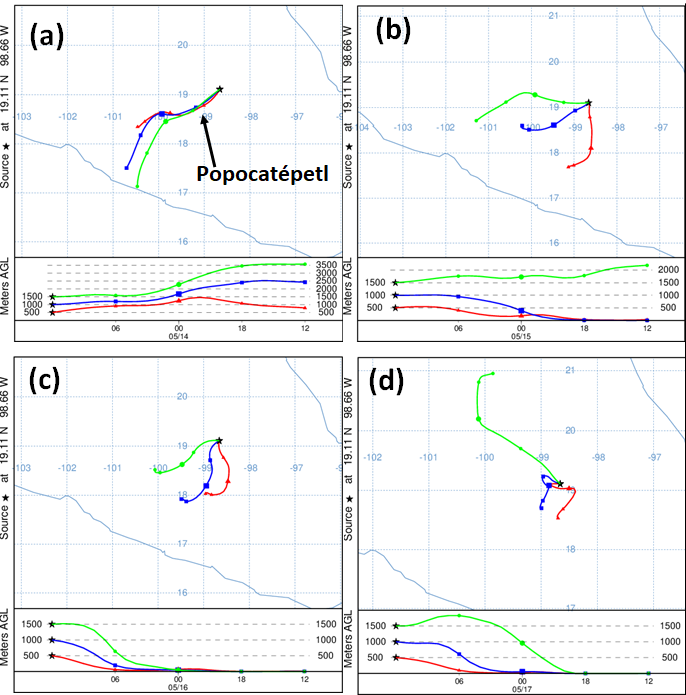


Fig. S7. 24-h backward air masses arriving at the AAO at different heights (100, 500, and 1000 above ground level) for May (a) 13, (b) 14, (c) 16, and (d) 17, 2019.

**Supplementary Tables**

**Table S1**. Global Horizontal Irradiance (GHI) and anomaly values for the VPAQ and PAQ days of May 2019.

|  | GHI (Wh/m^2^) | Anomaly (%) |
| --- | --- | --- |
| VPAQ | 1041 | -7.81 |
| PAQ | 1129 | -0.03 |
